# Supplementary material for: Content-rich biological network constructed by mining PubMed abstracts
Source: BMC Bioinformatics. 2004 Oct 8;5:147. doi: 10.1186/1471-2105-5-147 (PMC528731; doi:10.1186/1471-2105-5-147)
Supplement: Additional File 2 — The original results of the above study (non-essential files are deleted to keep the file size under the limit set by BMC bioinformatics). [file 1471-2105-5-147-S2.bz2 › chilibotAdditionalFile2/dip10/376ID10713102E9789/html/NGFB_NTRK1.html]

 


 **NGFB** and **NTRK1** 
  
Found 1439 abstracts in PubMed, retrieved 10.  
 

 What does Google say? 
 PDF only 
| .edu only 

---

**Interactive relationship** (e.g. stimulation, inhibition, etc)

**Inhibitory relationship**- The p75 NTR transfection only resulted in the mild differentiation response, and transfection of TrkA  [ **NTRK1** ]  gene caused remarkable neurite extension, up regulation of neurofilament and decreased expression of N myc oncogene after NGF  [ **NGFB** ]  treatment.  Ref: 12877805 Chin Med J (Engl), 2003
**Neutral relationship**- Degrees of down regulation in NGF  [ **NGFB** ]  family were greater than those in their receptors Trk  [ **NTRK1** ]  family.  Ref: 12854135 World J Gastroenterol, 2003
- On the other hand, the up regulated astroglial TrkA  [ **NTRK1** ]  receptor may be an important target for exogenous NGF  [ **NGFB** ] , which, as previously described J Neurosci 11 1991 2914.  Ref: 12849738 Neuroscience, 2003

**Non-interactive relationship** (e.g. studied together, co-existance, homology, etc.)

- NGF  [ **NGFB** ] , BDGF, NT 3, NT 4 5, NT 6 and TrkA  [ **NTRK1** ] , B and C were down regulated simultaneously in gastric carcinoma in comparison with normal gastric mucosa.  Ref: 12854135 World J Gastroenterol, 2003
- Both of NGF  [ **NGFB** ]  family and their receptors Trk  [ **NTRK1** ]  family mRNA were expressed in gastric cancer and normal gastric mucosa.  Ref: 12854135 World J Gastroenterol, 2003
- Here, we provide in vivo and in vitro evidence for the presence within DRG neurons of endosomes containing NGF  [ **NGFB** ] , activated TrkA  [ **NTRK1** ] , and signaling proteins of the Rap1 Erk1 2, p38MAPK, and PI3K Akt pathways.  Ref: 12848933 Neuron, 2003
- In particular, the putative role of NGF  [ **NGFB** ]  and its receptor TrkA  [ **NTRK1** ]  in immune system homeostasis has become a much studied topic.  Ref: 12892403 J Anat, 2003
- CONCLUSION Down regulated expression of NGF  [ **NGFB** ]  family and their receptors Trk  [ **NTRK1** ]  family mRNA in gastric cancer is confirmed.  Ref: 12854135 World J Gastroenterol, 2003
- Western blot analysis confirmed these results, showing that the lacrimal gland expresses NGF  [ **NGFB** ] , BDNF, NT3, and NT4 as well as the NTrs TrkA  [ **NTRK1** ] , TrkB, and TrkC and the p75 protein.  Ref: 12882781 Invest Ophthalmol Vis Sci, 2003
- RT PCR showed positive bands at the appropriate sizes for NGF  [ **NGFB** ] , BDNF, NT3, and NT4, and for the receptors TrkA  [ **NTRK1** ] , TrkB, TrkC, and p75.  Ref: 12882781 Invest Ophthalmol Vis Sci, 2003
- To determine which of the neurotrophins NTs nerve growth factor  [ **NGFB** ]  NGF  [ **NGFB** ] , brain derived neurotrophic factor BDNF, neurotrophin 3 NT3, and neurotrophin 4 5 NT4 and their receptors NTrs, TrkA  [ **NTRK1** ] , TrkB, TrkC, and p75, are present in the adult rat lacrimal gland.  Ref: 12882781 Invest Ophthalmol Vis Sci, 2003
- METHODS Retroviral vectors were constructed to express the high affinity NGF  [ **NGFB** ]  receptor TrkA  [ **NTRK1** ]  and low affinity NGF  [ **NGFB** ]  receptor p75 NTR.  Ref: 12877805 Chin Med J (Engl), 2003
- We have examined the effect of global transient cerebral ischemia, evoked in rat by 10 min of cardiac arrest, upon the changes in the cellular expression of two nerve growth factor  [ **NGFB** ]  NGF  [ **NGFB** ]  receptors TrkA  [ **NTRK1** ]  and p75 in the hippocampus.  Ref: 12849738 Neuroscience, 2003
- The cotransfection of the two genes into this cell line resulted in the more rapid and more apparent morphological changes than single TrkA  [ **NTRK1** ]  transfected cells after NGF  [ **NGFB** ]  treatment.  Ref: 12877805 Chin Med J (Engl), 2003
- However, NGF  [ **NGFB** ]  rescue from growth factor withdrawal requires NGF  [ **NGFB** ]  signalling through the high affinity tyrosine kinase receptor  [ **NTRK1** ]  TrkA  [ **NTRK1** ] , while NGF  [ **NGFB** ]  rescue from TNFalpha promoted cell death requires NGF  [ **NGFB** ]  signalling through the low affinity p75NTR receptor.  Ref: 12882321 Aging Cell, 2003
- OBJECTIVE To assess the effects of both TrkA  [ **NTRK1** ]  and p75 NTR on nerve growth factor  [ **NGFB** ]  NGF  [ **NGFB** ]  induced differentiation of neuroblastoma cells.  Ref: 12877805 Chin Med J (Engl), 2003
- Quantitative in situ hybridization was used to detect changes in neuronal PACAP mRNA expression and to correlate this expression with neuronal mRNA expression of the NGF  [ **NGFB** ]  receptor tyrosine kinase trk A  [ **NTRK1** ] .  Ref: 12890505 Neuroscience, 2003
- NGF  [ **NGFB** ]  family and their receptors Trk  [ **NTRK1** ]  family probably play a unique role in gastric cancer cell apoptosis by a novel Ras or Raf signal transduction pathway.  Ref: 12854135 World J Gastroenterol, 2003
